# Idiopathic multiple castleman disease case combined with severe neuropathy, Sjogren’s syndrome and membrane nephropathy treated by rituximab: a case report and literature review

**DOI:** 10.3389/fimmu.2026.1804654

**Published:** 2026-06-05

**Authors:** Shu Wang, Tong Chen, Shaojun Liu, Zunguo Du, Yanyan Kong, Yan Yuan, Tianling Ding, Qian Wang

**Affiliations:** 1 Department of Hematology, Huashan Hospital, Fudan University, Shanghai, China; 2 Department of Nephrology, Huashan Hospital, Fudan University, Shanghai, China; 3 Department of Pathology, Huashan Hospital, Fudan University, Shanghai, China; 4 Department of Nuclear Medicine and PET Center, Huashan Hospital, Fudan University, Shanghai, China

**Keywords:** idiopathic multicentric castleman disease, iMCD-NOS, membranous nephropathy, rituximab, Sjögren’s syndrome, VEGF

## Abstract

**Background:**

Idiopathic multicentric Castleman disease (iMCD) is a lymphoproliferative disorder characterized by dysregulated systemic immunity. Multiple cytokines had been found involved in the disease pathogenesis. Hence, involvement of multiple systems in iMCD complicates diagnosis and efficacy assessments. Although guidelines recommend anti-interleukin-6 (IL-6) agents as the primary treatment, options for second-line therapy remain indeterminate.

**Case presentation:**

A 65-year-old woman presented with progressive polyneuropathy and nephrotic-range proteinuria ten days after COVID-19 vaccination. Evaluation revealed multicentric lymphadenopathy, elevated IL-6, and plasmacytic-variant CD histopathology (HHV-8 negative). Concurrent Sjögren’s syndrome and anti-PLA2R-negative membranous nephropathy were confirmed. After exclusion of POEMS syndrome, iMCD-NOS with intermediate severity was diagnosed. Initial rituximab-cyclophosphamide-dexamethasone therapy resulted in paradoxical neurological worsening despite declining VEGF levels. Anti-IL-6 therapy was inaccessible due to economic constraints. Single-agent rituximab was initiated and continued for nine cycles over 24 months, achieving clinical remission by January 2024 with near-normalization of inflammatory markers, resolution of proteinuria, and neurological recovery.

**Conclusions:**

This case demonstrates that rituximab monotherapy can achieve clinical remission in iMCD-NOS with concurrent autoimmune manifestations when anti-IL-6 therapy is unavailable. The delayed response pattern—with biomarker improvement preceding clinical recovery—highlights the importance of serial VEGF monitoring and persistence with B-cell–directed therapy before concluding treatment failure.

## Introduction

Castleman disease (CD) comprises a group of heterogeneous lymphoproliferative disorders that share common histopathological features but exhibit distinct pathologies, clinical manifestations, and outcomes. Multicentric CD (MCD) is subdivided into HHV-8–associated MCD, POEMS-associated MCD, and idiopathic MCD (iMCD), the latter defined by the absence of HHV-8 infection and monoclonal plasma cell proliferation. Three clinical subtypes of iMCD are recognized: iMCD-TAFRO (thrombocytopenia, anasarca, fever, renal dysfunction, and organomegaly), iMCD-IPL (idiopathic plasmacytic lymphadenopathy, featuring thrombocytosis and hypergammaglobulinemia), and iMCD-NOS (not otherwise specified) (1, 2).

Recent pathological findings suggest that iMCD may be an inflammatory cytokine syndrome involving multiple systems, driven by dysregulated proliferation of B cells and other lineages (3, 4). iMCD is frequently associated with autoimmune manifestations, with approximately 22.5% of patients having concurrent autoimmune diseases and 47% testing positive for connective tissue disorder–associated autoantibodies (5). Renal involvement, including membranous nephropathy, and polyneuropathy without POEMS syndrome are recognized features of iMCD (6, 7).

Due to the mechanism of a dysregulated cytokine storm, anti-interleukin-6 (IL-6) targeted agents are recommended as first-line therapy for iMCD (8). Monoclonal antibodies targeted against IL-6, Siltuximab, or against the IL-6 receptor, Tocilizumab, were used (8, 9). However, anti-IL-6 agents were not accessible due to economic and/or area-related factors. Moreover, patients with disease refractory to anti-IL-6 targeted agents were also observed (7). Currently, there is no consensus on alternative treatment options when anti-IL-6 therapy is ineffective. A recent finding uncovered that activated B cells might be the main source of IL-6 in iMCD (10). Regimens including corticosteroids, rituximab, immunosuppressant, or systemic chemotherapy could be tried (11).

## Case report

**Figure 1:**
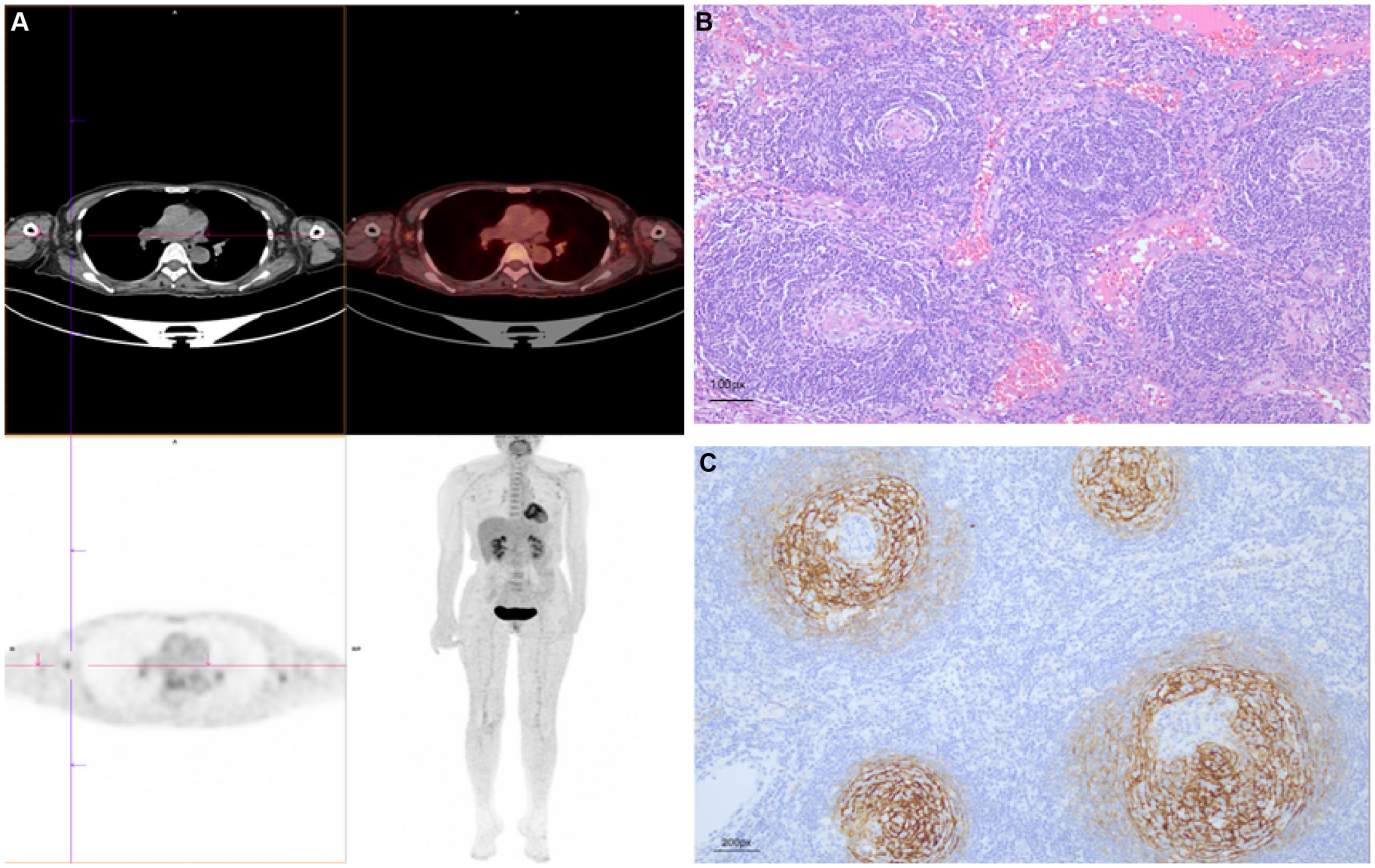
**(A)** Multiple lymphadenopathies found by PETCT scan. **(B)** Hematoxylin-eosin (HE) stained pathological photomicrograph of a lymph node magnified 100 times. **(C)** CD20+ B cells immunohistology staining of the lymph node.

**Figure 2:**
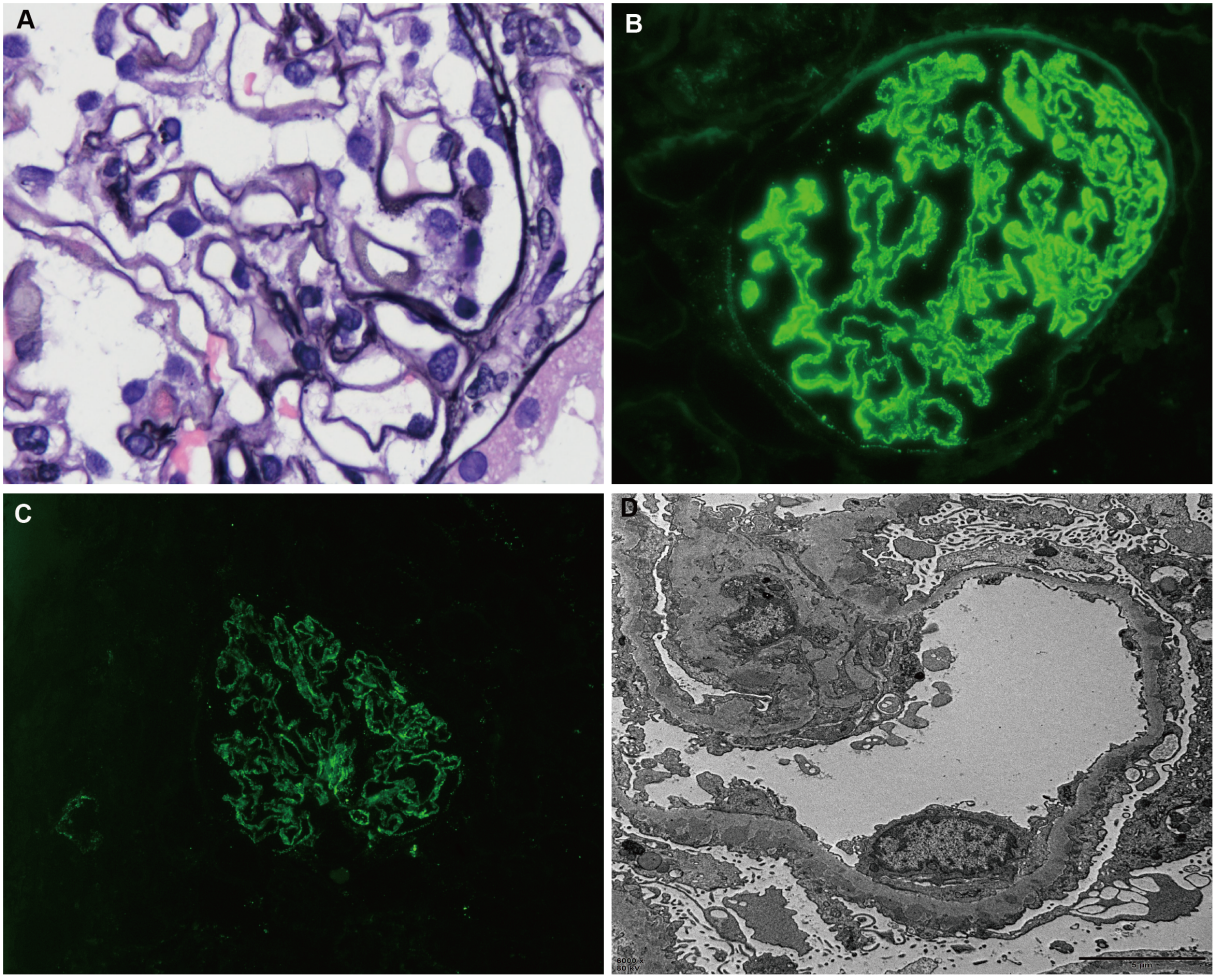
**(A)** Light microscopy (1000×): Silver staining reveals thickening of the glomerular basement membrane, with numerous vacuolization visible. **(B, C)** Immunofluorescence (200×): Fine granular deposits are observed along the capillary walls, with positive staining for the IgG1 subclass **(D)** Electron microscopy (6000×): Numerous subepithelial electron-dense deposits are observed, accompanied by mild reactive proliferation of the basement membrane and prominent effacement of foot processes.

A 65-year-old woman, previously in good health, presented with frothy urine and progressive limb weakness with numbness beginning ten days after coronavirus disease 2019 (COVID-19) vaccination in June 2021. Over the ensuing two months, her symptoms worsened progressively, with functional decline to an Eastern Cooperative Oncology Group (ECOG) performance status of 2 and a Neuropathy Impairment Score (NIS) of 14 (**Table 1**). Electromyography revealed a mixed polyneuropathy with both demyelinating and axonal features affecting motor and sensory nerves. A comprehensive panel of peripheral neuropathy–associated antibodies was negative. Serum immunoglobulin levels demonstrated hypogammaglobulinemia (IgG 5.05 g/L, IgA 1.88 g/L, IgM 0.19 g/L, and IgG4 0.398 g/L; IgG4/IgG ratio 7.88%). Inflammatory markers were discordant, with an elevated interleukin-6 (IL-6) level of 5.85 pg/ml and erythrocyte sedimentation rate (ESR) of 72 mm/h, whereas C-reactive protein (CRP) was 0.5 mg/L. Serum albumin was low at 23 g/L. Complete blood count remained normal on serial testing. Autoimmune serologies revealed elevated antinuclear antibody (ANA, 1:320), anti-Ro52 (40), anti-SSA (62), and anti-SSB (99), despite the absence of sicca symptoms. Given the serological profile, a labial salivary gland biopsy was performed, which demonstrated focal acinar atrophy with lymphoplasmacytic interstitial infiltration, consistent with Sjögren’s syndrome. Ultrasound examination revealed multiple lymphadenopathies involving the cervical, supraclavicular, and axillary regions, without hepatosplenomegaly. To evaluate for additional autoimmune or malignant disease, positron emission tomography–computed tomography (PET-CT) was performed, demonstrating multiple mildly hypermetabolic lymphadenopathies (SUVmax 2.1) and reactive bone marrow changes without sclerotic lesions (**Figure 1A**). Excisional biopsy of an axillary lymph node revealed regressed follicular centers with atrophic germinal centers, a whorled (“onion-skin”) pattern of the mantle zone, prominent vascular proliferation with hyalinized vessels penetrating into follicles, and scattered plasma cell infiltration in the interfollicular areas. Immunohistochemistry demonstrated abundant IgG4-positive plasma cells (>40/HPF) with an IgG4/IgG ratio of 40%. *In situ* hybridization for Epstein-Barr virus–encoded RNA (EBER) was negative, and immunohistochemical staining for HHV-8 was negative. These findings were consistent with CD, plasmacytic variant (**Figures 1B, C**). Serum and urine immunofixation electrophoresis revealed no monoclonal proteins or free light chains. Bone marrow biopsy demonstrated normal hematopoiesis without monoclonal plasma cell proliferation. No significant endocrine abnormalities, organomegaly, or skin changes were identified. Vascular endothelial growth factor (VEGF) was not measured prior to treatment initiation. Serological testing for HIV, Epstein-Barr virus (EBV), HHV-8, tuberculosis, syphilis, and other relevant pathogens was uniformly negative. Twenty-four-hour urine protein quantification was 3.08 g. Kidney biopsy demonstrated anti-phospholipase A2 receptor (anti-PLA2R) antibodies were negative, combined with occasional segmental subendothelial deposition of loose plasma protein-like material, indicating membranous nephropathy, Stages I–II, which was considered secondary to the underlying systemic disease (**Figure 2**). Based on the constellation of multicentric lymphadenopathy with characteristic plasmacytic-variant histopathology, elevated IL-6, polyneuropathy, secondary membranous nephropathy, and concurrent Sjögren’s syndrome—after exclusion of HHV-8 infection, POEMS syndrome, and other known causes of multicentric CD—a diagnosis of iMCD, not otherwise specified (iMCD-NOS) with an intermediate severity score (iMCD International Prognostic Index, iMCD-IPI) was established in September 2021 (9).

**Table 1:** Summary of laboratory and clinical variations.

Timepoint	VEGF (pg/ml)	IL-6 (pg/ml)	Proteinuria (g/24h)	ECOG	NIS
Baseline (Aug 2021)	—	5.85	3.08	2	14
Oct 2021 (post-RCD)	872	—	5.5	4	64
Nov 2021 (ICD)	321	2.41	3	4	123
Dec 2021 (ICD)	144	8.98	4.28	3	108
Jul 2022 (3 cycles R)	206	3.31	0.2	2	54
Jan 2024 (9 cycles R)	123	2.82	0	1	12

**Figure 3:**
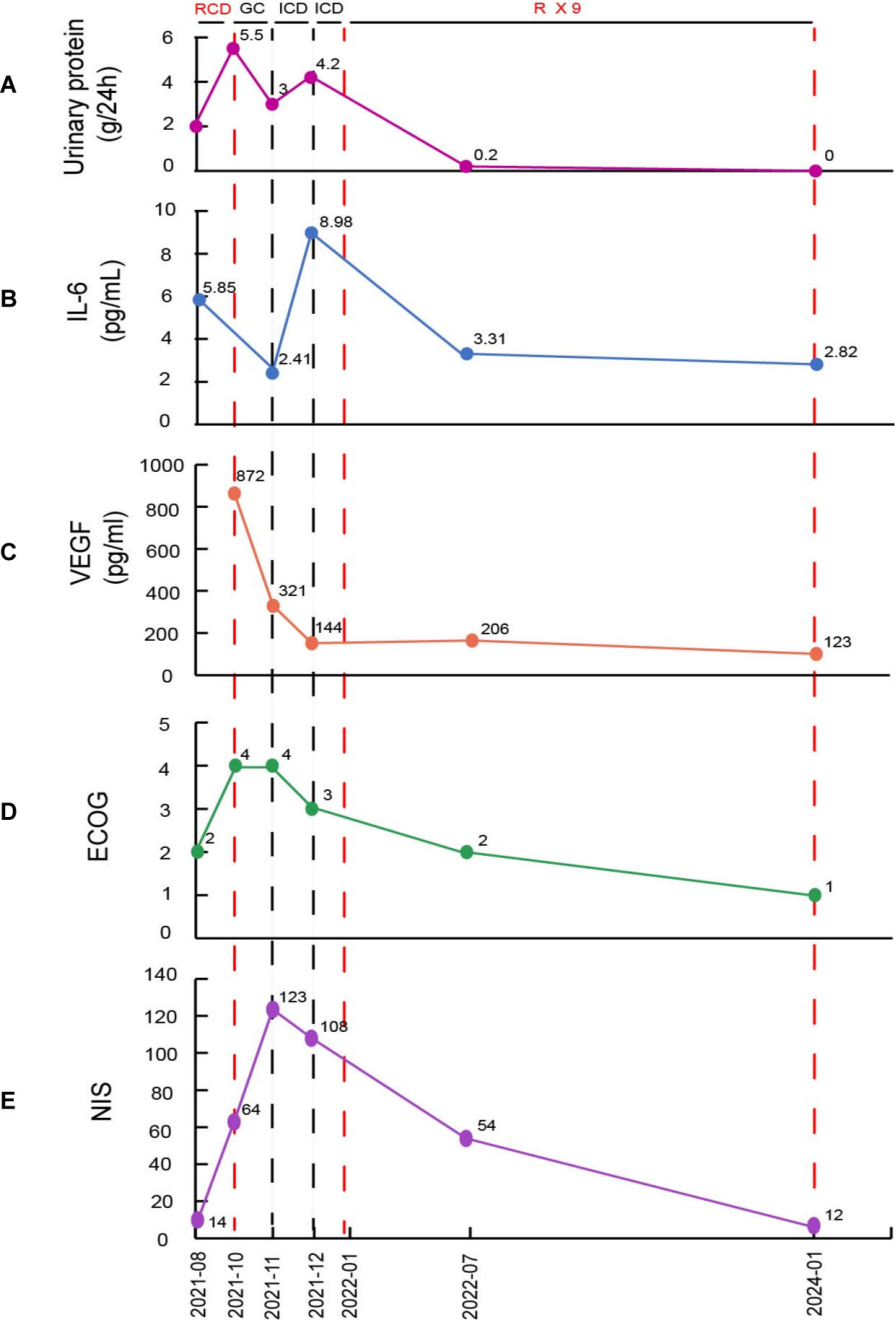
Four-line graph demonstrated the dynamics level of urinary protein [**(A)**, pink], IL-6 [**(B)**, blue], VEGF [**(C)**, orange], ECOG [**(D)**, green], and NIS [**(E)**, purple] throughout the course of treatment. Each indicator and the corresponding unit were labeled on the vertical axis of each panel. Time point was labeled on the horizonal axis. Dot lines represented the time points for each treatment or assessment. Each treatment regimen was labeled on the top of dot lines. ECOG, Eastern Cooperative Oncology Group; GC, glucocorticosteroid; ICD, Ixazomib plus cyclophosphamide and dexamethasone; IL-6, interleukin 6; NIS, Neuropathy Impairment Score; R, Rituximab; RCD, Rituximab plus cyclophosphamide and dexamethasone; VEGF, vascular endothelial growth factor.

Initial therapy was commenced on 16 September 2021, consisting of intravenous immunoglobulin (IVIg, 0.4 g/kg daily for 5 days) followed by a combined regimen of rituximab (500 mg), cyclophosphamide (1 g), and dexamethasone (15 mg daily for 5 days) (RCD regimen). Despite this intervention, the patient’s neurological status deteriorated progressively over the ensuing weeks, manifesting as inability to ambulate or perform fine motor movements (ECOG 4, NIS 64). Serum VEGF, measured in early October 2021, was markedly elevated at 872 pg/ml (**Figure 3**). On 18 October 2021, pulse methylprednisolone (80 mg, Day 1) was administered, followed by a 7-day tapering course of oral prednisone. Anti-IL-6 agents (siltuximab or tocilizumab), recommended as first-line therapy for HHV-8–negative iMCD per international consensus guidelines, were considered but were inaccessible due to economic constraints (1). Although inflammatory markers subsequently declined (IL-6 2.41 pg/ml; VEGF 321 pg/ml), neurological symptoms continued to worsen (NIS 123). Given the refractory neuropathy, a proteasome inhibitor–based regimen was initiated in November 2021, comprising ixazomib (4 mg), cyclophosphamide (400 mg), and dexamethasone (20 mg, Days 1–2) (ICD regimen). Two times of ICD were administered between November and December 2021; however, the regimen was discontinued due to severe and immediate gastrointestinal toxicity. Notably, neurological symptoms showed early improvement during this period, with decreased limb numbness and weakness (ECOG 3). Laboratory evaluation at the end of the ICD regimen revealed a discordant biomarker pattern: IL-6 had risen to 8.98 pg/mL, and 24h urine protein had increased to 4.28 g, whereas VEGF had continued to decline to 144.96 pg/ml (**Figure 1**). The NIS at this timepoint was 108, reflecting the cumulative neurological deficit despite emerging clinical improvement. In light of the sustained decline in VEGF levels—suggesting a delayed but ongoing response to the initial rituximab administration—the treatment strategy was revised to single-agent rituximab (600 mg). The first dose was administered on 1 January 2022, approximately four months after the prior rituximab infusion. Subsequent treatment intervals were extended beyond the planned schedule due to financial constraints.

After three cycles of single-agent rituximab, assessment in July 2022 demonstrated concordant improvement across all disease domains: VEGF had decreased to 206 pg/ml, IL-6 to 3.31 pg/ml, 24h urine protein to 0.2 g, and neurological function had substantially recovered (ECOG 2, NIS 54). The patient continued to improve clinically over the subsequent 18 months, although comprehensive laboratory evaluation per CD Collaborative Network (CDCN) response criteria was not performed at every assessment (10). Upon completion of the ninth cycle of rituximab in January 2024, the patient had achieved durable clinical remission, as evidenced by near-normalization of inflammatory markers (VEGF 123 pg/ml, IL-6 2.82 pg/ml) and neurological function (ECOG 1, NIS 12). The total treatment duration from initiation to last rituximab infusion was approximately 28 months.

## Discussion

This case illustrates several clinically important aspects of iMCD not otherwise specified (iMCD-NOS), including the diagnostic challenges posed by overlapping autoimmune conditions, the complexity of treatment sequencing when first-line anti-IL-6 therapy is unavailable, and the phenomenon of delayed response to rituximab-based therapy.

The diagnosis of iMCD was established on 2017 consensus criteria, fulfilling both major criteria (characteristic lymph node histopathology and multicentric lymphadenopathy) and multiple minor criteria (elevated ESR, hypoalbuminemia, elevated IL-6, and proteinuria) (1, 11). The plasmacytic histopathological variant, mildly elevated IL-6 (5.85 pg/ml), and absence of thrombocytopenia or anasarca distinguished this case from iMCD-TAFRO. The iMCD-IPL subtype was considered given the abundant IgG4+ cells (>40/HPF). However, this patient lacked the defining IPL features: serum IgG was 5.05 g/L (not elevated), IgM was suppressed, and platelet count was normal rather than elevated (1, 12). The histopathologic features—follicular atrophy with vascular implantation and whorled mantle zone—are more characteristic of hyaline-vascular morphology, whereas iMCD-IPL typically demonstrates sheetlike plasmacytosis with hyperplastic germinal centers (13). The iMCD-NOS classification is therefore appropriate.

IgG4-RD was excluded based on a serum IgG4/IgG ratio of 7.88% (below the 19% cutoff discriminating IgG4-RD from iMCD), tissue IgG4/IgG of 40%, and absence of characteristic organ involvement (12, 14, 15). The elevated IgG4+ cells likely reflect IL-6-driven polyclonal B-cell activation inherent to iMCD (12). Notably, 33% of iMCD-IPL cases meet histological criteria for IgG4-RD due to numerous IgG4+ plasma cells, particularly when serum IgG exceeds 5000 mg/dl (12). The key distinguishing features include: IgG4-RD demonstrates a high IgG4: IgG ratio, allergic features, and germinal center expansion involving T follicular helper cells, iMCD involves polyclonal antibody production (high IgA and IgM levels), sheet-like mature plasma cell proliferation, and inflammatory features driven by IL-6 (14).

POEMS syndrome was excluded by the absence of the mandatory criterion—monoclonal plasma cell dyscrasia—with negative immunofixation electrophoresis and no clonal plasma cells on bone marrow biopsy (11). No sclerotic bone lesions, skin changes, or clinically significant endocrinopathy were present. The NCCN guidelines specify that POEMS is considered a disease “associated” with CD, and because the monoclonal plasma cells are believed to drive the cytokine storm, it is classified as “POEMS-associated MCD” rather than iMCD (1).

The 2017 iMCD criteria list specific autoimmune diseases as exclusions but explicitly state that “detection of autoimmune antibodies alone is not exclusionary.” Sjögren’s disease is not among the listed exclusion criteria. The lip biopsy was prompted by positive serologies (anti-SSA, anti-SSB) rather than sicca symptoms; although without xerophthalmia or xerostomia, the patient still meets 2016 ACR-EULAR classification criteria for Sjögren’s disease. It’s reported up to 30% of Sjögren’s disease patients lack sicca symptoms (16). These serological findings may reflect polyclonal B-cell hyperreactivity driven by iMCD. A recent study found that 47% of iMCD patients tested positive for at least one connective tissue disorder-associated autoantibody, including those associated with Sjögren’s syndrome (7). Autoimmune diseases have been reported in 22.5% of CD patients, including SS specifically (17). A case remarkably similar to ours—iMCD with concurrent SS and secondary membranous nephropathy—has been reported by Pan et al., demonstrating that these conditions can coexist (6). The simultaneous improvement of all manifestations with rituximab suggests shared B-cell-driven pathophysiology rather than two independent disease processes.

The anti-PLA2R-negative membranous nephropathy is consistent with secondary membranous nephropathy (MN) associated with iMCD rather than primary MN or Sjögren’s-associated nephropathy (which is typically tubulointerstitial rather than glomerular) (18). Glomerular nephropathy is specifically listed as a disorder associated with iMCD in the consensus diagnostic criteria (1). Although renal complications of Castleman’s disease more commonly include AA amyloidosis, thrombotic microangiopathy, and membranoproliferative glomerulonephritis, membranous nephropathy has been reported (18).

Recent research has revealed subtype-specific differences in IL-6 production: in iMCD-NOS/IPL, plasma cells are the predominant IL-6-expressing cells, whereas in iMCD-TAFRO, vascular endothelial cells express IL-6 (13). This distinction has therapeutic implications, as plasma cell-predominant IL-6 production may explain favorable responses to IL-6 blockade therapy in iMCD-IPL, while elevated serum IL-6 in TAFRO may be a secondary phenomenon of the cytokine storm (13). Spatial and single-cell mapping studies have identified CXCL13+ follicular dendritic cells (FDCs), PDGFRA+ T-zone reticular cells (TRCs), and ACTA2+ perivascular reticular cells (PRCs) as predominant sources of increased VEGF expression and IL-6 signaling in CD (19). VEGF promotes cell survival, angiogenesis, and vascular permeability; it could account for the hypervascularization in lymph nodes, extravascular fluid overload, and eruptive cherry hemangiomatosis observed in patients with iMCD (20). Rituximab targets this cytokine cascade by depleting CD20+ B cells, thereby interrupting the supply of new plasma cells and reducing IL-6/VEGF production (21). This mechanism explains why B-cell depletion can be effective even when IL-6 levels are only mildly elevated, as in this case.

Rituximab is an established second-line treatment option in current NCCN guidelines for non-severe iMCD when siltuximab and tocilizumab are not available (1). A Chinese cohort study demonstrated a 55.5% overall response rate (CR 33.3%, PR 22.2%) with rituximab-containing regimens and a 5-year OS of 81% (22). The initial RCD regimen coincided with neurological deterioration, likely reflecting insufficient time for B-cell depletion rather than treatment failure, as rituximab’s mechanism requires weeks to months for full effect.

Ixazomib was selected as bridge therapy to target the plasma cell compartment during delayed rituximab efficacy. This approach is analogous to bortezomib-based regimens, which are listed in NCCN guidelines as alternative regimens for subsequent therapy. The rationale was based on the understanding that plasma cells are the predominant IL-6 source in iMCD-NOS/IPL subtypes (13). A recent study demonstrated that rituximab-bortezomib-dexamethasone (RVD) induces high response rates in iMCD, with all five enrolled patients achieving PR or better (23). However, in our case, ICD was discontinued due to gastrointestinal toxicity.

The decision to continue rituximab monotherapy was guided by progressively declining VEGF levels, suggesting an ongoing delayed response despite initial clinical deterioration. VEGF is listed as a supportive feature in the iMCD diagnostic criteria and is useful for monitoring therapy (1). The temporal dissociation between biomarker improvement and neurological recovery reflects the slow kinetics of axonal regeneration after inflammatory control is achieved. Rituximab was administered as a finite nine-cycle course, representing a practical advantage over siltuximab, which requires continuous indefinite infusions (24). The NCCN guidelines support this approach, noting that after response to rituximab-based time-limited therapy, observation is appropriate, with the option to repeat rituximab ± prednisone without limit if progression occurs ≥6 months after completion. This approach reduces long-term treatment burden, which is particularly relevant in resource-limited settings where anti-IL-6 agents may be inaccessible.

The temporal relationship between COVID-19 vaccination and symptom onset is reported descriptively. While case reports have documented new-onset iMCD and TAFRO syndrome following COVID-19 vaccination, a causal relationship cannot be established from individual cases (25, 26). COVID-19 vaccination has been associated with various autoimmune and autoinflammatory phenomena, potentially through molecular mimicry, bystander activation, or epitope spreading. However, the rarity of iMCD and the widespread administration of COVID-19 vaccines make it difficult to distinguish true causation from coincidental temporal association.

Several limitations should be acknowledged. Firstly, the observed improvement may reflect cumulative effects of sequential therapies (IVIG, RCD, corticosteroids, ICD, rituximab monotherapy); the efficacy of rituximab monotherapy should be interpreted cautiously. Furthermore, a key limitation of this report is that serial evaluations did not consistently include all parameters required by the CDCN consensus response criteria. While the available clinical and laboratory data demonstrate sustained and meaningful improvement, the absence of systematic CDCN response assessment at each time point limits the ability to formally categorize the depth of response. Notably, this case may suggest that rituximab is a potential alternative when anti-IL-6 therapy is unavailable, but it does not establish efficacy. Prospective studies are needed to confirm these observations. Additionally, the mildly elevated IL-6 (5.85 pg/ml) and normal CRP are unusual for iMCD and warrant consideration of alternative diagnoses. However, cytokine and proteomic profiling has revealed normal IL-6 levels in many patients with iMCD, and some patients with low IL-6 levels respond to anti-IL-6 therapy while some with high levels do not.

## Conclusion

This case illustrates the diagnostic complexity of iMCD-NOS, particularly the mandatory differential diagnosis with iMCD-IPL, IgG4-RD, and POEMS syndrome. The serum IgG4/IgG ratio (7.88%, below the 19% cutoff) and absence of characteristic IgG4-RD organ involvement were critical discriminators. The coexistence of Sjögren’s-associated serologies likely reflects polyclonal B-cell hyperreactivity rather than true primary Sjögren’s disease, as supported by the absence of sicca symptoms and the simultaneous improvement of all manifestations with B-cell-directed therapy.

The achievement of clinical remission with rituximab monotherapy, guided by VEGF monitoring despite initial apparent treatment failure, highlights the importance of biomarker-guided treatment decisions and patience with B-cell-directed therapy. The finite-duration rituximab approach offers a practical advantage in resource-limited settings. Further prospective studies are needed to define optimal treatment strategies for iMCD-NOS, particularly when first-line anti-IL-6 therapy is unavailable.

## Data Availability

The original contributions presented in the study are included in the article/supplementary material. Further inquiries can be directed to the corresponding author/s.
